# Level-Set Method for Image Analysis of Schlemm's Canal and Trabecular Meshwork

**DOI:** 10.1167/tvst.9.10.7

**Published:** 2020-09-04

**Authors:** Xin Wang, Yuxi Zhai, Xueyan Liu, Wei Zhu, Jianlu Gao

**Affiliations:** 1 Department of Ophthalmology, Liaocheng People's Hospital, Cheeloo College of Medicine, Shandong University, Liaocheng, Shandong, China; 2 Department of Ophthalmology, Liaocheng People's Hospital, Liaocheng, Shandong, China; 3 Department of Mathematics, Liaocheng University, Liaocheng, Shandong, China; 4 Department of Pharmacology, Qingdao University School of Pharmacy, Qingdao, Shandong, China; 5 Qingdao Haier Biotech Co. Ltd, Qingdao, Shandong, China

**Keywords:** image segmentation, ultrasound biomicroscopy, Schlemm's canal, trabecular meshwork, intraocular pressure

## Abstract

**Purpose:**

To evaluate different segmentation methods in analyzing Schlemm's canal (SC) and the trabecular meshwork (TM) in ultrasound biomicroscopy (UBM) images.

**Methods:**

Twenty-six healthy volunteers were recruited. The intraocular pressure (IOP) was measured while study subjects blew a trumpet. Images were obtained at different IOPs by 50-MHz UBM. ImageJ software and three segmentation methods—*K*-means, fuzzy C-means, and level set—were applied to segment the UBM images. The quantitative analysis of the TM-SC region was based on the segmentation results. The relative error and the interclass correlation coefficient (ICC) were used to quantify the accuracy and the repeatability of measurements. Pearson correlation analysis was conducted to evaluate the associations between the IOP and the TM and SC geometric measurements.

**Results:**

A total of 104 UBM images were obtained. Among them, 84 were adequately clear to be segmented. The level-set method results had a higher similarity to ImageJ results than the other two methods. The ICC values of the level-set method were 0.97, 0.95, 0.9, and 0.57, respectively. Pearson correlation coefficients for the IOP to the SC area, SC perimeter, SC length, and TM width were −0.91, −0.72, −0.66, and −0.61 (*P* < 0.0001), respectively.

**Conclusions:**

The level-set method showed better accuracy than the other two methods. Compared with manual methods, it can achieve similar precision, better repeatability, and greater efficiency. Therefore, the level-set method can be used for reliable UBM image segmentation.

**Translational Relevance:**

The level-set method can be used to analyze TM and SC region in UBM images semiautomatically.

## Introduction

Glaucoma is the world's second-leading ocular disease that causes blindness and is the primary cause of irreversible blindness.^1^ Elevated intraocular pressure (IOP) is harmful to the optic nerve and can aggravate glaucoma. Therefore, IOP is the most widely used parameter for evaluating and monitoring glaucoma.^2^ IOP is balanced by the production and outflow of the aqueous humor. Most studies on glaucoma pathogenesis have focused on outflow resistance. The trabecular meshwork (TM) and Schlemm's canal (SC) pathway account for 75% to 80% of the whole outflow,^3^ making it an important area for study.

Kagemann et al.^4^ showed that an acute IOP elevation can reduce the SC area and alter the TM configuration in human and animal eyes.^5^ Yan et al.^6^ demonstrated that aerobic exercise can cause TM and SC expansion, which lowers IOP. These findings suggest that TM-SC tissue configurations may determine aqueous outflow and IOP regulation. This conclusion also applies to patients with glaucoma. Swain et al.^7^ reported that SC is collapsed in most patients with primary open-angle glaucoma (POAG). Moreover, clinical studies have shown that canaloplasty is an effective and safe procedure to lower IOP in patients with POAG.^8^ Cagini et al.^9^^,^^10^ determined that canaloplasty is not as successful in eyes that exhibit an irreversible collapse of outflow pathways. These findings suggest that morphologic changes of TM-SC in patients with glaucoma can lower IOP and improve the disease.

Since TM-SC morphologic changes can influence IOP regulation, identifying a means to monitor the changes in this area has become an important goal in glaucoma research. Imaging systems are emerging as influential tools for evaluating the TM-SC region in vivo. Optical coherence tomography (OCT) and ultrasound biomicroscopy (UBM) are two of the most predominantly used imaging methods in ophthalmology. The former is considered powerful in distinguishing TM on account of its higher resolution and noninvasive nature.^11^^,^^12^ The latter can be used in almost all kinds of patients, even those who have a cloudy cornea or arcus senilis and cannot be examined with OCT.

Image segmentation is an important task in medical analysis and a critical step in many clinical applications. In addition, different clustering methods are used for medical image segmentation, such as *K*-means clustering^13^ and fuzzy C-means clustering (FCM).^14^ In previous studies, UBM image segmentation was performed freehand or partly assisted by image analysis software.^6^^,^^15^^–^^18^ Among them, ImageJ software (National Institutes of Health, Bethesda, MD) was the most commonly used. However, manual segmentation is time-consuming and depends on the experience of the technician. Moreover, UBM images are usually corrupted with intensity inhomogeneities, which make TM and SC segmentation an inherently difficult task.^19^ Recently, level-set methods have been proposed to deal with images with intensity inhomogeneity, such as the local intensity clustering method^20^^,^^21^ and the edge-based method.^22^ These approaches have been successfully applied to segment magnetic resonance images of the breast, X-ray images of bones, ultrasound images of the prostate,^20^ and infrared breast thermography.^23^

Inspired by the above research, we investigated the application of different segmentation methods for UBM images. Trumpet playing has the advantages of convenience, feasibility, and simplicity. Therefore, we adopted trumpet playing as an intervention to obtain IOP elevations and fluctuations.^24^ These segmentation methods were further verified in UBM images obtained under different IOPs. With these segmentation results, quantitative analysis of the TM-SC region was done and compared.

## Methods

### Image Acquisition

In this study, healthy volunteers were recruited from the staff of Liaocheng People's Hospital, Shandong Province, China. All subjects underwent an ophthalmologic examination and were verified to have met the following requirements: (1) 20 to 40 years old, (2) IOP between 10 and 21 mm Hg, (3) normal anterior chamber depth and open angle, (4) normal structure of TM examined by a gonioscope, (5) no history of inflammatory eye disease or eye surgery, and (6) no family history of glaucoma. The whole process was approved by the ethics committee of Liaocheng People's Hospital and adhered to the tenets of the Declaration of Helsinki. Written informed consent was obtained from each subject before the study began. Participants were examined in a supine position while blowing the trumpet. They were asked to blow the trumpet slowly for as long as possible.^24^ One eye of each participant was randomly selected for the UBM examination using a 50-MHz UBM (Suoer SW-3200L; Suowei Co., Tianjin, China). The IOP of the other eye before and during the trumpet blowing was measured using the Icare Pro tonometer (Tiolat Oy, Helsinki, Finland). According to the results of our preexperiment and previous studies,^6^ the SC detection rate at the inferior quadrant is the highest. Thus, all the images were obtained from the inferior quadrant of the eye at four time points: before trumpet blowing, 10 seconds after the start of blowing (IOP increasing period), immediately after blowing cessation (IOP peak time), and 10 seconds after blowing cessation. All examinations were conducted by the same ophthalmologists and under the same illumination conditions using identical equipment.

### Algorithms

Image segmentation is the process of partitioning a digital image into multiple regions. In this study, three algorithms were assessed: *K*-means,^13^ FCM,^14^ and the level-set method.^20^

### *K*-Means Clustering

*K*-means^13^ is a fast and simple clustering algorithm that is used to classify an image into a specific number of disjointed clusters. The general idea is to identify *K* centroids, one for each cluster, and then associate each data point to the nearest centroid.^25^ Let Ω be the image domain, *I*(*x*): Ω → *R* be the observed image, and *x*, *y* represent the pixel coordinates. In a previous study,^26^ segmentation of image *I*(*x*) into *K* clusters was achieved by minimizing the following equation:

(1) $$E=\sum_{j=1}^{K}\sum_{i=1}^{M}{I\left(x_{i}^{\left(j\right)}\right)-c_{j}}^{2},$$

where $∥I\left(x_{i}^{\left(j\right)}\right)-c_{j}∥$ is the distance between one of the pixels, $I(x_{i}^{\left(j\right)})$, in cluster *j* and its cluster centroid, *c_j_*, and *M* is the pixel number of the image.

### FCM Algorithm

The FCM algorithm was first suggested by Dunn^27^ and later improved by Bezdek et al.^14^ This algorithm is widely used in data clustering and image segmentation.^28^^,^^29^ By introducing the possibility of partial memberships to clusters, this algorithm attempts to partition every pixel into a collection of fuzzy cluster centroids by minimizing the following objective function:

(2) $$E=\sum_{j=1}^{K}\sum_{i=1}^{M}u_{{}_{ij}}^{m}{∥I\left(x_{i}^{\left(j\right)}\right)-c_{j}∥}^{2},$$

where *u_ij_* is the fuzzy membership degree of pixel $I(x_{i}^{\left(j\right)})$ and cluster centroid *c_j_*, which satisfies *u_ij_* ∈ [0, 1], and $\sum_{i=1}^{M}u_{ij}=1,j=1,2,\cdots ,K$. In addition, parameter *m*(*m* > 1) is a constant that determines the fuzziness of the resulting partitions.

### Level-Set Method

From the physics of imaging, the observed UBM image *I* can be modeled as

(3) I=BJ+n,

where *J*(*x*) is the real image, *B*(*x*) is the bias field that accounts for the intensity inhomogeneity, and *n*(*x*) is the noise term.^30^ The bias field *B*(*x*) is assumed to change slowly, and the value *B*(*x*) can be considered approximately constant in a neighborhood of *O_y_* = {*x*||*x* − *y*| ≤ ρ}, *B*(*x*) ≈ *B*(*y*) for *x* ∈ *O_y_*. Real image *J* reflects an intrinsic property of the imaging objects, which can be assumed to be a piecewise constant. Moreover, *J* takes approximately *N* distinct constant values *c*_1_,*c*_2_,⋅⋅⋅*c_N_* in disjointed regions Ω_1_,Ω_2_,⋅⋅⋅Ω_*N*_, where $\Omega =\cup_{i=1}^{N}\Omega_{i}$ and Ω_*i*_∩Ω_*j*_ = Ø for *i* ≠ *j*. Thus, the intensities of points in each subregion Ω_*i*_∩*O_y_* can be approximated as follows:

(4) $$I\left(x\right)\approx B\left(y\right)c_{i}+n\left(x\right)\mathrm{for}x\in \Omega_{i}\cap O_{y}$$

Based on the assumption of zero-mean additive Gaussian noise, the intensities in neighborhood *O_y_* can be classified into *N* distinct clusters with centers *m_i_* ≈ *B*(*y*)*c_i_*:

(5) $$I_{y}^{i}=\left\{I\left(x\right):x\in \Omega_{i}\cap O_{y}\right\},i=1,2,\cdots ,N.$$

The *K*-means method is used to classify the local intensities in *O_y_*. Then, clustering criterion function ε_*y*_ of *y* in Ω can be written as

(6) $${ɛ}_{y}=\sum_{i=1}^{N}\int_{\Omega_{i}}k\left(y-x\right){\left|I\left(x\right)-B\left(y\right)c_{i}\right|}^{2}dx$$

where *k*(*y* − *x*) is the Gaussian kernel function, which is selected as a truncated Gaussian function defined by^25^

(7) $$k\left(y-x\right)=\left\{\begin{array}{cc} \frac{1}{a}e^{-\frac{{\left|y-x\right|}^{2}}{2\sigma^{2}}}, & x\in O_{y},\\ 0, & x\notin O_{y} \end{array}\right.$$

where *a* is a normalization constant, such that ∫*k*(*u*)*du* = 1, and σ is the standard deviation of the function. The smaller the value of ε_*y*_, the better the classification of *y* in Ω. Therefore, the optimal partition of the entire domain Ω can be realized by joint-minimizing ε_*y*_, which can be written as the following local clustering criterion function:

(8) $$\begin{array}{c} \begin{array}{c} ɛ=\int \;{ɛ}_{y}dy=\int \;\;\left(\sum_{i=1}^{N}\int_{\Omega_{i}}k(y-x){\left|I\left(x\right)-B\left(y\right)c_{i}\right|}^{2}dx\;\right)\;dy. \end{array}\;\;\; \end{array}$$

However, function ε is difficult to solve. Therefore, ε is converted into a level-set formulation with several level-set functions. Let φ: Ω → *R* be a level-set function, and function ε can be written as the function of Φ = (φ_1_,φ_2_,⋅⋅⋅φ_*k*_), *c* = (*c*_1_,*c*_2_,⋅⋅⋅*c_N_*) and the bias field *b*:

(9) $$ɛ\left(\Phi ,c,b\right)=\int \sum_{i=1}^{N}\int_{\Omega_{i}}e_{i}\left(x\right)m_{i}\left(\Phi \left(x\right)\right)dx,$$

where *e_i_*(*x*) = ∫*k*(*y* − *x*)|*I*(*x*) − *B*(*y*)*c_i_*|^2^*dy* and the membership functions *m_i_*=1 for *y* ∈ Ω_*i*_, and *m_i_*= 0 for *y*∉Ω_*i*_. For the case of two phases, the membership functions are defined by *m*_1_(φ) = *H*(φ) and *m*_2_(φ) = 1 − *H*(φ). The energy function in the two-phase level-set formulation is defined by

(10) $$F\left(\phi ,c,b\right)=ɛ\left(\phi ,c,b\right)+\nu L\left(\phi \right)+\mu R_{p}\left(\phi \right)$$

where *L*(φ) and *R_p_*(φ) are the regularization terms. Energy minimization is achieved by an iterative process. By minimizing this energy, the level-set method^30^ can segment the image and estimate the bias field that can be applied for bias correction. When the above energy function *F*(φ, *c*, *b*) obtains the minimum value or the maximum number of iterations is reached, the iteration is terminated.

Using ImageJ software, each image was segmented three times by the same ophthalmologist. The average value of the measurements was regarded as the ground truth. The paired *t-*test was used to compare the mean differences when the measurement data were obtained by ImageJ and the three segmentation methods (*P* *<* 0.05 was considered statistically significant).^31^^,^^32^ The relative error and the interclass correlation coefficient (ICC) were employed to quantify the accuracy and repeatability of the three methods.^33^ Because systematic differences are part of the measurement error, a two-way random-effects model was used to calculate the ICC.^34^

Image segmentation and analysis were conducted on images with clear TM-SC structures that were identified by the ophthalmologist. Owing to the low resolutions of the 50-MHz UBM images, it is almost impossible to obtain perfect quality UBM images. If the TM-SC region in the UBM image was primarily not deformed or slightly deformed but not significantly different from the physiologic anatomical structure, then the UBM image was considered of good quality and of use for segmentation. If the TM-SC region in the UBM image was severely deformed, was very different from the physiologic anatomy, or could not be recognized by the ophthalmologist, then the UBM image was considered of poor quality and excluded. Four UBM images of one subject were used to illustrate the segmentation effects of the different methods. The TM-SC regions of all UBM images were extracted using the above three segmentation methods. By measuring the TM-SC area, the reliability and repeatability of the segmentation results were quantified. Furthermore, the correlations between the measurements and the IOP were obtained. All experiments were carried out in MATLAB (MathWorks, Natick, MA) on a PC with a 3.6-GHz Intel core processor and 8 GB of memory.

## Results

**Figure 1.**
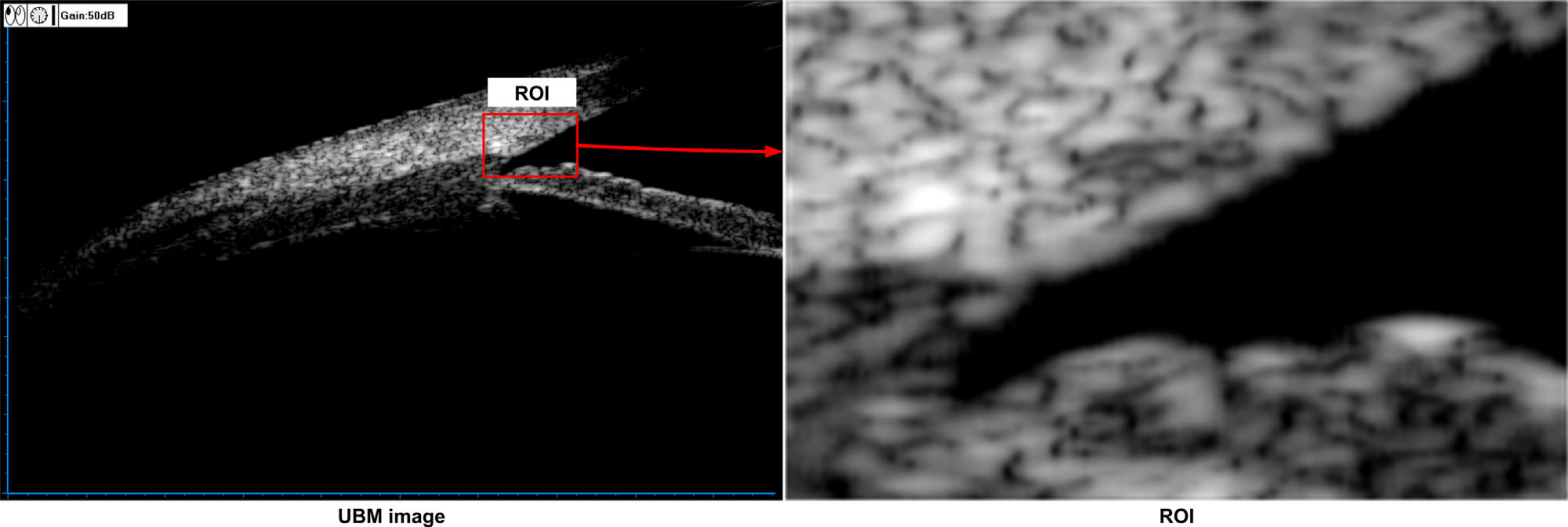
UBM image and ROI.

A total of 26 volunteers completed the whole experimental process, including 10 males and 16 females. The average age was 34.53 years. Four UBM images were collected from each subject at different IOPs, and 104 images were collected in total. The average IOP before trumpet blowing and immediately after was 17.5 mm Hg and 28.8 mm Hg, respectively. The UBM image and its region of interest (ROI) are shown in Figure 1. The size of the UBM image was 1024 × 655 pixels, and the size of the ROI was 150 × 100 pixels.

### Image Segmentation with Different Methods

**Figure 2.**
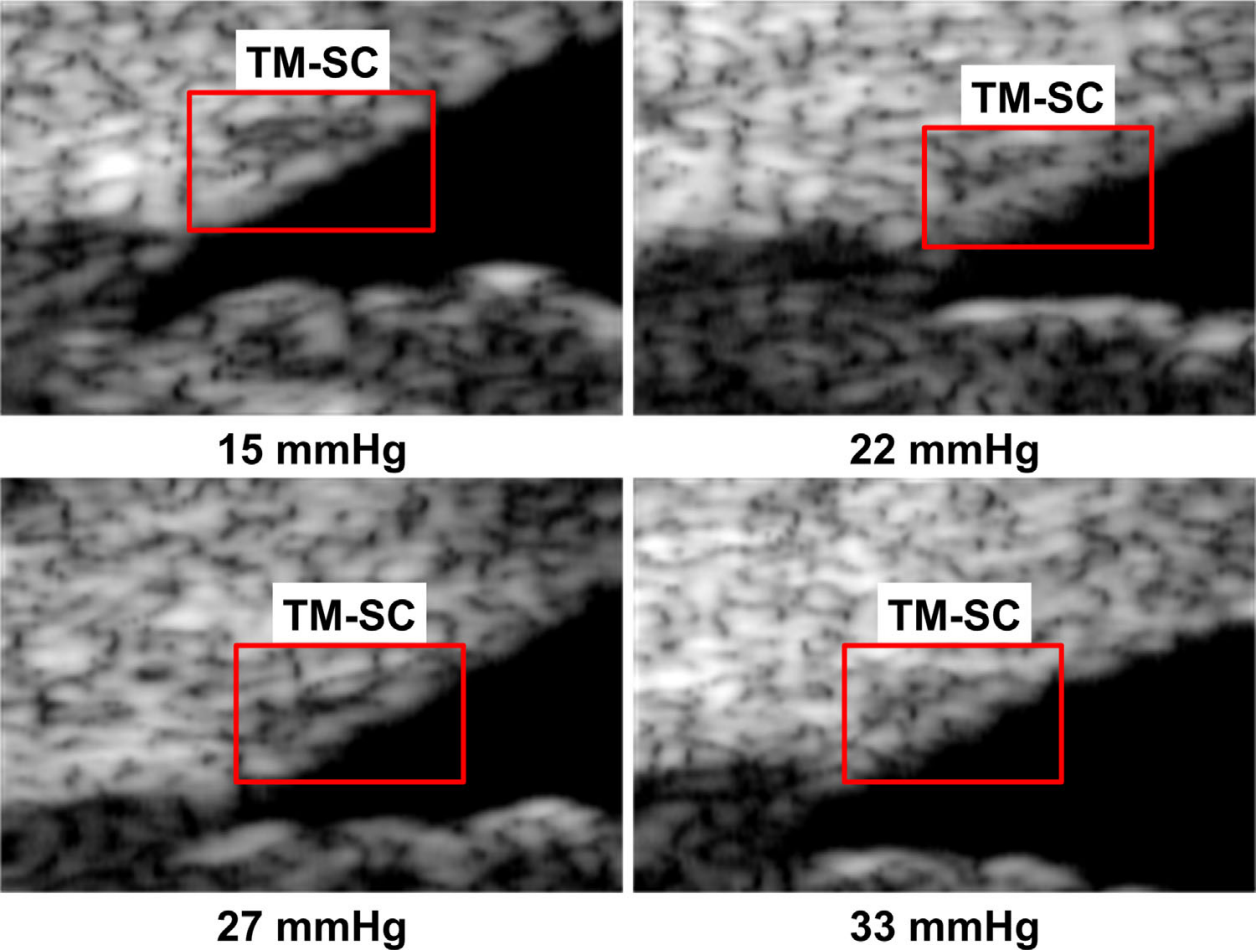
Preprocessed UBM images from one subject.

**Figure 3.**
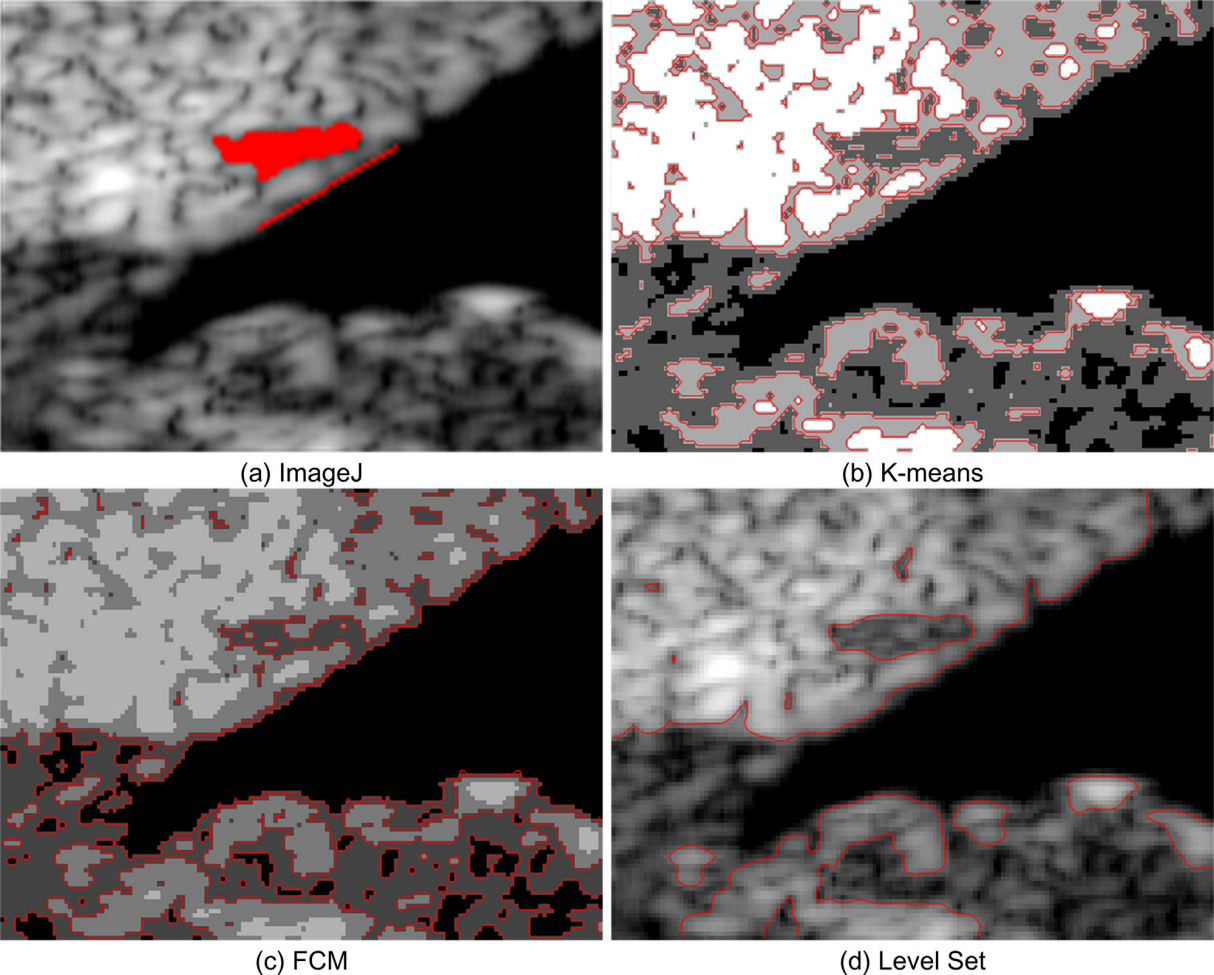
UBM image segmentation result of the ROI at the IOP of 15 mm Hg using (a) ImageJ, (b) *K*-means, (c) FCM, and (d) level set.

**Figure 4.**
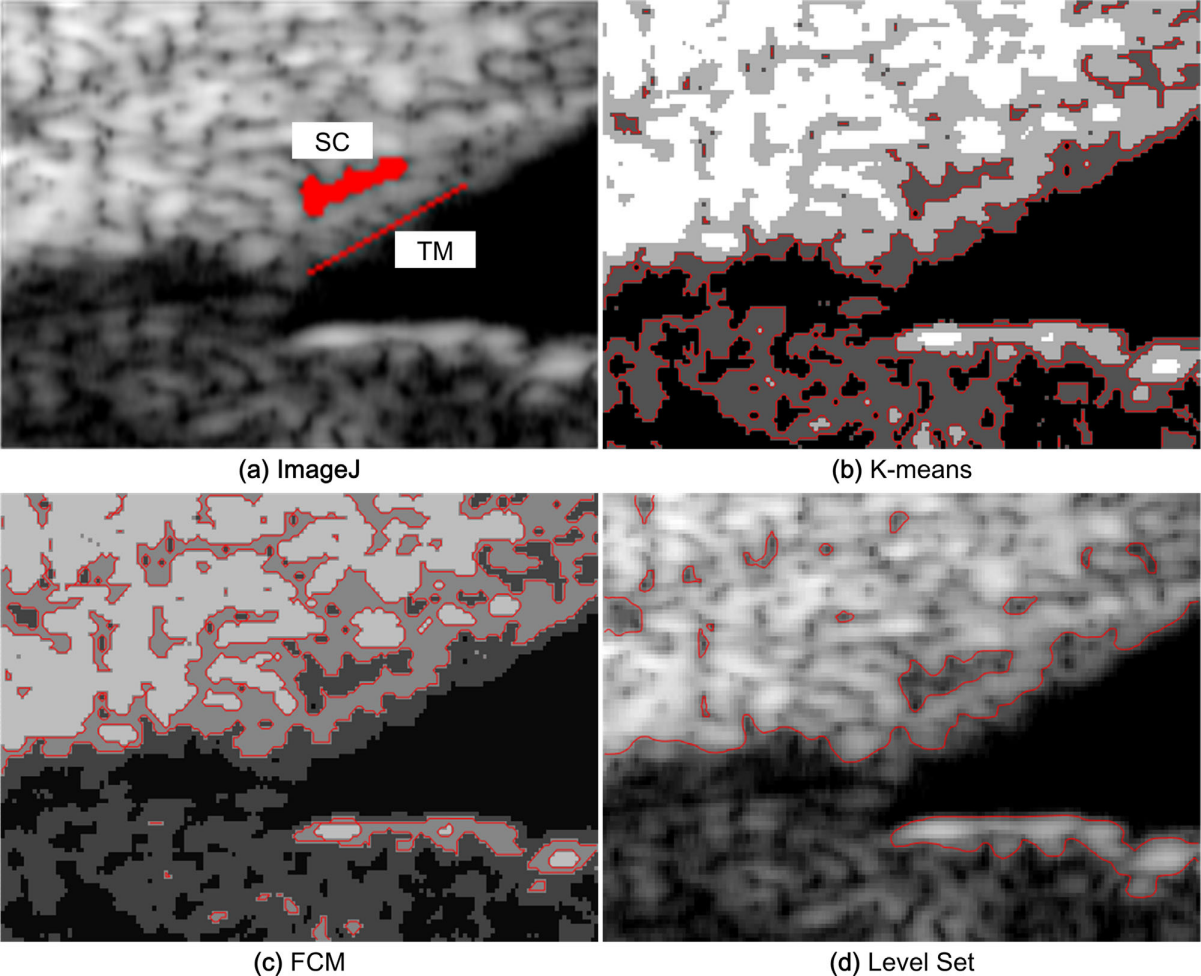
UBM image segmentation result of the ROI at the IOP of 22 mm Hg using (a) ImageJ, (b) *K*-means, (c) FCM, and (d) level set.

**Figure 5.**
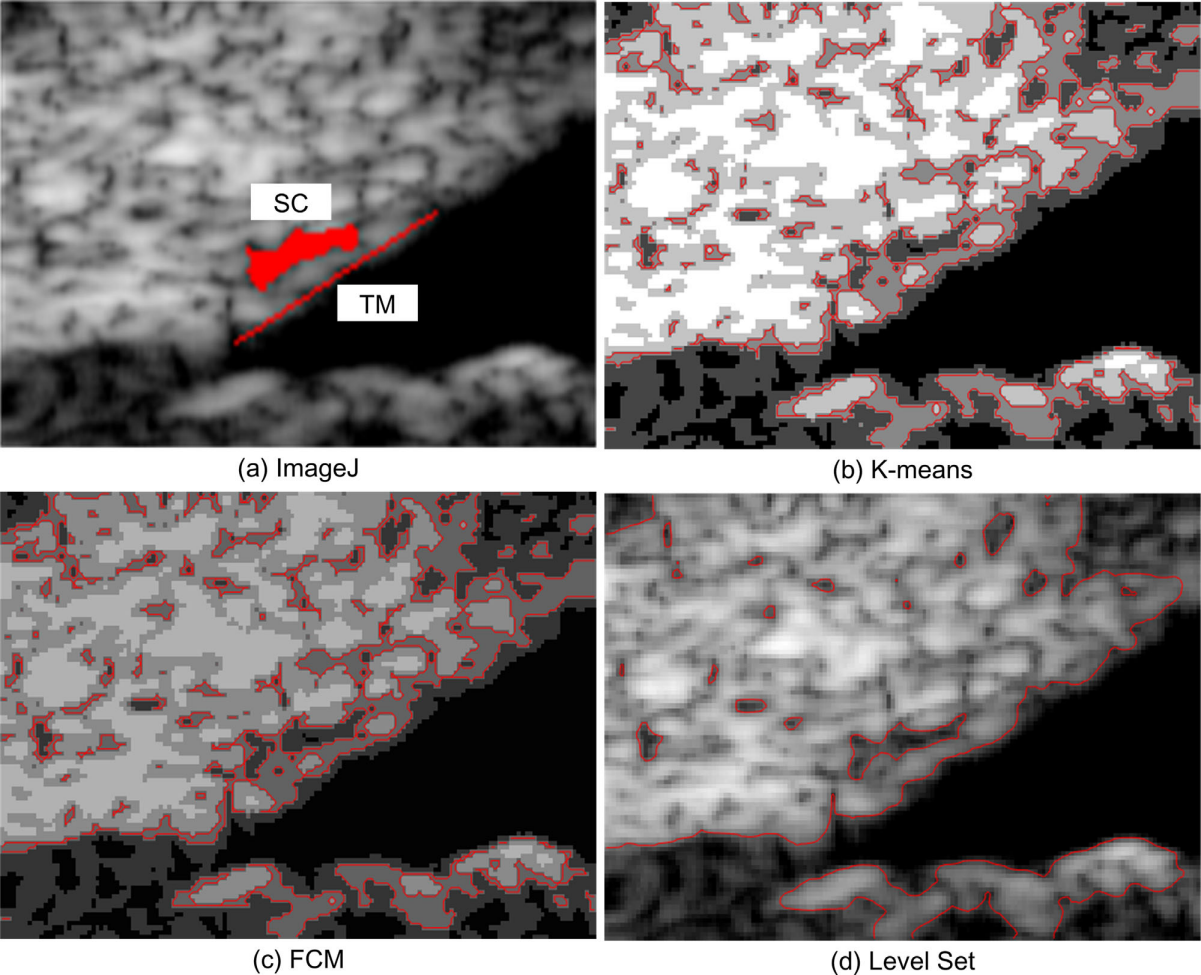
UBM image segmentation result of the ROI at the IOP of 27 mm Hg with (a) ImageJ, (b) *K*-means, (c) FCM, and (d) level set.

**Figure 6.**
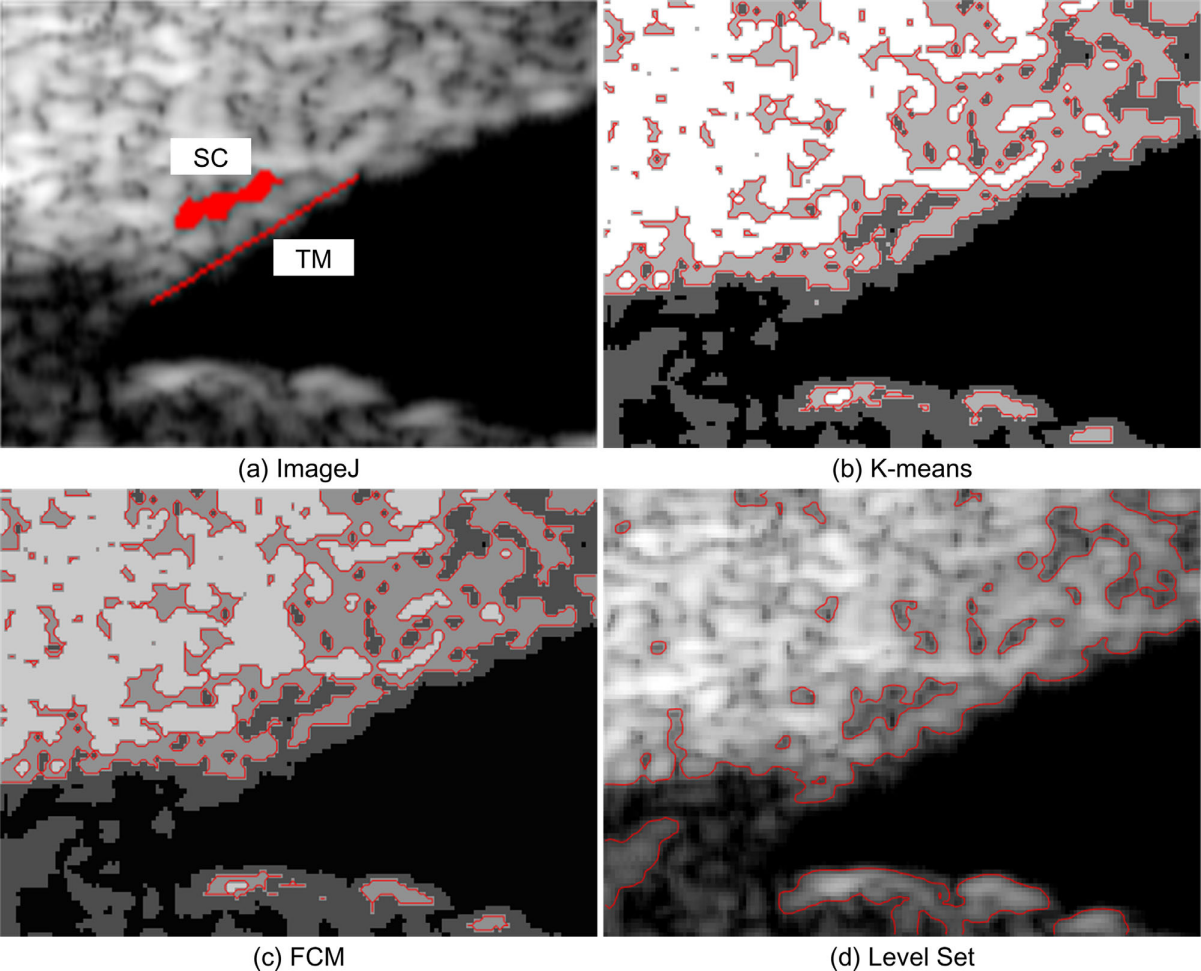
UBM image segmentation result of the ROI at the IOP of 33 mm Hg using (a) ImageJ, (b) *K*-means, (c) FCM, and (d) level set.

To illustrate the segmentation effects of the different methods, four UBM images (Fig. 2) from one subject were segmented. ImageJ, *K*-means, FCM, and level set were used to obtain the boundary curves of the different gray regions in the UBM images. The segmentation results are shown in Figures 3 to 6.

When the IOP is 15 mm Hg, the SC region segmentation result in Figure 3d is similar to that in Figure 3a. The TM and SC in Figures 3b and 3c are blended, resulting in an unrecognizable SC boundary, whereas the TM boundary in Figure 3d is easy to identify. The TM boundary in Figure 3b is discontinuous, and the boundary in Figure 3c is overlapped and unclear. In terms of the TM and SC segmentation effects in the UBM image, the level-set method is thus better than the other two methods.

Figure 4 shows the UBM image segmentation results of the ROI at the pressure of 22 mm Hg. It is observed that the SC region in Figure 4d is more consistent with Figure 4a. The SC boundaries in Figures 4b and 4c are rougher than those in Figure 4a. There are some disturbances on the TM boundary in Figure 4d. However, there are two TM boundaries in Figure 4b, and it is difficult to choose the correct one. The TM boundary in Figure 4c is oversegmented. Therefore, we can conclude that the level-set method can obtain results similar to those of ImageJ when the IOP is elevated.

The UBM image segmentation results at the pressure of 27 mm Hg are shown in Figure 5. The SC boundaries in Figures 5b and 5c obtained by the FCM and *K*-means methods are discontinuous. Some interference occurs between the TM and SC, which makes identifying the TM area difficult. The SC region segmented using the level-set method has a clear outline, and the TM region boundary can be easily identified. It is obvious that the level-set method produces more accurate segmentation results than the other two methods.

To verify the universality of the segmentation methods, we tested the three segmentation methods using the UBM image at the pressure of 33 mm Hg. From Figure 6, we can observe that the geometric deformation of SC becomes more obvious as the IOP increases. There are some small differences in the geometry of the TM-SC area in Figures 6a and 6d. The TM and SC in Figures 6b and 6c are connected on account of speckle noise, which also leads to the interruption of the TM boundary. The TM-SC region extracted by the level-set method is more similar to that of the ImageJ result than the other two methods.

### Quantitative Analysis of Segmentation Results

Eighty-four images that were adequately clear were selected from a total of 104 images. After the UBM images were segmented, the TM-SC region was extracted and measured. The measurement was performed as follows. The Canny edge detection algorithm was applied to convert the segmentation result to a binary form. The number of pixels inside the SC region was used as the SC area, and the number of pixels on the boundary was regarded as the SC perimeter. The average of the three maximum numbers of pixels per row in the SC region was used as the SC length. The Sobel edge detection algorithm was employed to binarize the boundary curve of the TM-SC region. The average of the three maximum numbers of pixels between the TM and SC per column was used as the TM width.

Table shows the measurement data of the four methods as the mean ± SD. There were no statistically significant differences between the measurement data obtained by the level-set method and ImageJ (*P* = 0.663, *P* = 0.071, *P* = 0.755, and *P* = 0.117 for SC area, SC perimeter, SC length, and TM width, respectively). There were no statistically significant differences in the SC area and SC perimeter measurements between the *K*-means method and by ImageJ (*P* = 0.103 and *P* = 0.901 for SC area and SC perimeter, respectively), while the differences for the SC length and TM width between the two methods were statistically significant (*P* < 0.001 for SC length and TM width). There was no statistically significant difference between the SC area measured by the FCM method and the corresponding result measured by ImageJ (*P* = 0.662), while the differences in the SC perimeter, SC length, and TM width between the two methods were statistically significant (*P* = 0.032, *P* < 0.001, and *P* < 0.001 for SC perimeter, SC length, and TM width, respectively).

**Table.** Measurement Data (Mean ± Standard Deviation) for Segmentation Results of the Four Methods (Pixels, *n* = 84)

Method	SC Area	SC Perimeter	SC Length	TM Width
ImageJ	150.23 ± 41.8	66.11 ± 16.35	22.17 ± 5.39	11.14 ± 0.97
Level set	150.71 ± 45.93	64.61 ± 17.01	22.25 ± 5.25	10.95 ± 1.37
*K*-means	161.47 ± 70.48	66.86 ± 31.01	26.3 ± 9.28	10.18 ± 1.83
FCM	145.07 ± 59.74	56.37 ± 31.08	26.7 ± 7.97	9.31 ± 1.55

As can be seen from the above results, the level-set method showed better similarity to the ImageJ method than the FCM and *K*-means methods. Among the four parameters measured by the ImageJ and three segmentation methods, the SC area was the most consistent parameter.

**Figure 7.**
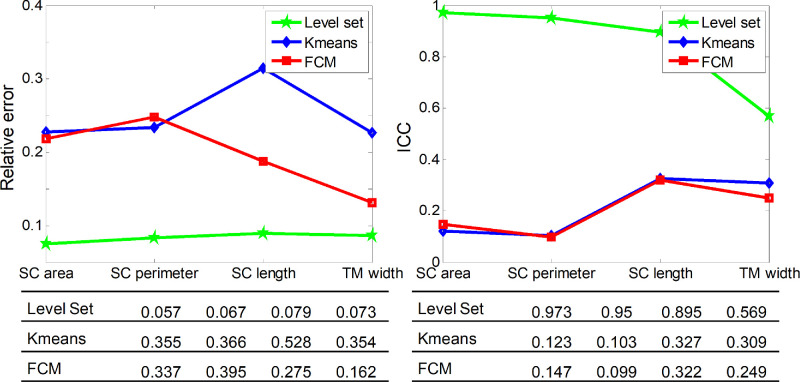
Schematic diagrams of relative errors and ICC values of the three segmentation methods.

Figure 7 presents schematic diagrams of the relative errors and ICC values of the three segmentation methods. It is evident that the relative errors of the level-set method are less than 0.08, whereas the other two methods have large relative errors. The ICC values of the level-set method are 0.97, 0.95, 0.9, and 0.57, respectively, whereas the corresponding ICC values of the other two methods are less than 0.4. From these results, we can conclude that the measurements of the level-set method have a higher reliability and better repeatability than the other two methods.

### Correlation Analysis Between IOP and Measurements

**Figure 8.**
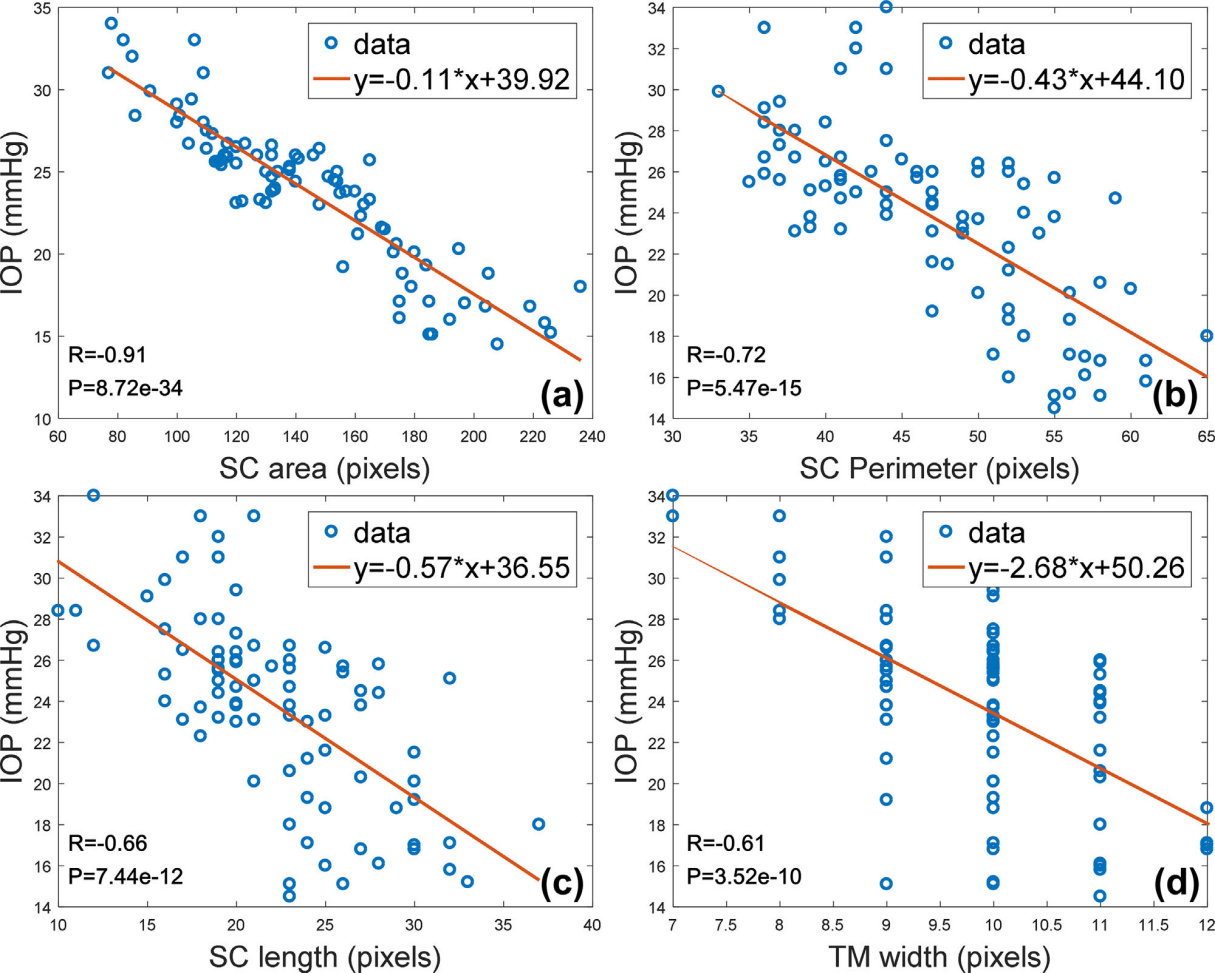
IOP versus measurements of the (a) SC area, (b) SC perimeter, (c) SC length, and (d) TM width. For reference, regression equations, linear regression lines (*red*), Pearson correlation coefficients (*R*), and *P* values are provided.

Owing to the poor performance of the *K*-means and FCM methods for segmenting the TM-SC region, only the level-set method was used to perform the correlation analysis. The correlations among the SC area, SC perimeter, SC length, TM width, and IOP were analyzed using the Pearson correlation coefficient and linear regression analysis. The results are shown in Figure 8. It can be observed that, as the IOP increases, the SC area, perimeter, and length tend to decrease. The TM width likewise decreases. Pearson correlation coefficients for IOP to the SC area, SC perimeter, SC length, and TM width are −0.91, −0.72, −0.66, and −0.61, respectively. Thus, a negative correlation relationship between the IOP and the geometrical measurement of TM and SC can be inferred.

## Discussion

Image segmentation plays an important role in many medical imaging applications. In previous studies, the TM-SC region was manually obtained from UBM images.^6^^,^^15^^–^^18^ However, manual outlining is a time-consuming and tedious task. In this article, we showed that the level-set method can be used to extract the TM-SC region from the UBM image and useful features can be obtained from the segmentation. Compared with manual segmentation, the level-set method produced similar segmentation results while providing better repeatability and efficiency. In addition, the level-set method had higher accuracy than the classical FCM and *K*-means methods.

The negative correlation between IOP and the measurements of TM and SC inferred from our results were similar to those of previous studies.^4^^,^^5^ The reason for TM-SC region collapse may be the compression force caused by acute IOP elevation to the elastic structure. Assuming that accurate measurements of the TM and SC response to IOP fluctuation in patients in vivo are realized, mathematical models can be used to calculate the TM stiffness, which has recently been shown to be associated with resistance to outflow.^35^^–^^37^ Among the four measurement indicators in this study, the Pearson correlation coefficient for IOP to the SC area is the highest. Thus, we may speculate that the SC area can be used as a sensitive indicator for measuring the TM-SC region.

However, our study had the following limitations. First, the resolution of the 50-MHz UBM was not high; therefore, approximately 20% of the images could not be used. Second, since the UBM images were continuously obtained while the measurement of IOP was discontinuous, the mismatch between the two may have affected the correlation analysis results. Third, the subjects recruited for this experiment were young healthy adults. We are therefore unsure whether this method can be used with elder subjects and patients with glaucoma. Therefore, the effectiveness of the level-set method for UBM image segmentation of different subject groups will be our future work.

In conclusion, changes in the TM-SC region can be detected by UBM and extracted by image segmentation methods. The level-set method can accurately and efficiently segment UBM images of TM and SC. Therefore, the level-set method is an effective technique for UBM image segmentation.

## References

[1] Tham YC, Li X, Wong TY, Quigley HA, Aung T, Cheng CY Global prevalence of glaucoma and projections of glaucoma burden through 2040: a systematic review and meta-analysis. *Ophthalmology*. 2014; 121: 2081–2090. 24974815 10.1016/j.ophtha.2014.05.013

[2] Sihota R, Angmo D, Ramaswamy D, Dada T Simplifying “target” intraocular pressure for different stages of primary open-angle glaucoma and primary angle-closure glaucoma. *Indian J Ophthalmol*. 2018; 66: 495–505. 29582808 10.4103/ijo.IJO_1130_17 PMC5892050

[3] Grant WM Clinical measurements of aqueous outflow. *AMA Arch Ophthalmol*. 1951; 46: 113–131. 14856471 10.1001/archopht.1951.01700020119001

[4] Kagemann L, Wang B, Wollstein G, et al. IOP elevation reduces Schlemm's canal cross-sectional area. *Invest Ophthalmol Vis Sci*. 2014; 55: 1805–1809. 24526436 10.1167/iovs.13-13264 PMC3968930

[5] Battista SA, Lu Z, Hofmann S, Freddo T, Overby DR, Gong H Reduction of the available area for aqueous humor outflow and increase in meshwork herniations into collector channels following acute IOP elevation in bovine eyes. *Invest Ophthalmol Vis Sci*. 2008; 49: 5346–5352. 18515571 10.1167/iovs.08-1707 PMC4788035

[6] Yan X, Li M, Song Y, et al. Influence of exercise on intraocular pressure, Schlemm's canal, and the trabecular meshwork. *Invest Ophthalmol Vis Sci*. 2016; 57: 4733–4739. 27607419 10.1167/iovs.16-19475

[7] Swain DL, Ho J, Lai J, Gong H Shorter scleral spur in eyes with primary open-angle glaucoma. *Invest Ophthalmol Vis Sci*. 2015; 56: 1638–1648. 25670488 10.1167/iovs.14-15593 PMC4351652

[8] Riva I, Brusini P, Oddone F, Michelessi M, Weinreb RN, Quaranta L Canaloplasty in the treatment of open-angle glaucoma: a review of patient selection and outcomes. *Adv Ther*. 2019; 36: 31–43. 30488337 10.1007/s12325-018-0842-6 PMC6318242

[9] Cagini C, Peruzzi C, Fiore T, Spadea L, Lippera M, Lippera S Canaloplasty: current value in the management of glaucoma. *J Ophthalmol*, 2016; 2016: 1–6. 10.1155/2016/7080475 PMC4867063 27239337

[10] Zhang J, Wang NL Progression on canaloplasty for primary open angle glaucoma. *Int J Ophthalmol*. 2019; 12: 1629–1633. 31637200 10.18240/ijo.2019.10.16 PMC6796084

[11] Xin C, Johnstone M, Wang N, Wang RK OCT study of mechanical properties associated with trabecular meshwork and collector channel motion in human eyes. *PLoS One*. 2016; 11: e0162048. 27598990 10.1371/journal.pone.0162048 PMC5012558

[12] Hariri S, Johnstone M, Jiang Y, et al. Platform to investigate aqueous outflow system structure and pressure-dependent motion using high-resolution spectral domain optical coherence tomography. *J Biomed Opt*. 2014; 19: 106013. 25349094 10.1117/1.JBO.19.10.106013 PMC4210620

[13] Kanungo T, Mount DM, Netanyahu NS, Piatko CD, Silverman R, Wu A An efficient k-means clustering algorithm: analysis and implementation. *IEEE T Pattern Anal*. 2002; 24: 881–892.

[14] Bezdek JC, Ehrlich R, Full W FCM: The fuzzy c-means clustering algorithm. *ComputGeosci*. 1984; 10: 191–203.

[15] Gillmann K, Bravetti GE, Mermoud A, Mansouri K A prospective analysis of iStent inject microstent positioning: Schlemm canal dilatation and intraocular pressure correlations. *J Glaucoma*. 2019; 28: 613–621. 31058666 10.1097/IJG.0000000000001273

[16] Daniel MC, Dubis AM, Quartilho A, et al. Dynamic changes in Schlemm canal and iridocorneal angle morphology during accommodation in children with healthy eyes: a cross-sectional cohort study. *Invest Ophthalmol Vis Sci*. 2018; 59: 3497–3502. 30025073 10.1167/iovs.17-23189

[17] Fernández-Vigo JI, García-Feijóo J, Martínez-de-la-Casa JM, García-Bella J, Fernández-Vigo JA Morphometry of the trabecular meshwork in vivo in a healthy population using Fourier-domain optical coherence tomography. *Invest Ophthalmol Vis Sci*. 2015 ; 56: 1782–1788. 25698706 10.1167/iovs.14-16154

[18] Chen Z, Song Y, Li M, et al. Schlemm's canal and trabecular meshwork morphology in high myopia. *Ophthalmic Physiol Opt*. 2018; 38: 266–272. 29691920 10.1111/opo.12451

[19] Xiao G, Brady M, Noble JA, Zhang Y Segmentation of ultrasound B-mode images with intensity inhomogeneity correction. *IEEE T Med Imaging*. 2002; 21: 48–57. 10.1109/42.981233 11838663

[20] Li CM, Huang R, Ding ZH, Gatenby JC, Metaxas DN, Gore JC A level set method for image segmentation in the presence of intensity inhomogeneities with application to MRI. *IEEE T Image Process*. 2011; 20: 2007–2016. 10.1109/TIP.2011.2146190 PMC6952214 21518662

[21] Wang XF, Min H, Zhang YG Multi-scale local region based level set method for image segmentation in the presence of intensity inhomogeneity. *Neurocomputing*. 2015; 151: 1086–1098.

[22] Vasilevskiy A, Siddiqi K Flux-maximizing geometric flows. *IEEE T Pattern Anal*. 2002; 24: 1565–1578.

[23] Golestani N, Etehadtavakol M, Ng EYK Level set method for segmentation of infrared breast thermograms. *Excli J*. 2014; 13: 241–251. 26417258 PMC4464455

[24] Schmidtmann G, Jahnke S, Seidel EJ, Sickenberger W, Grein HJ Intraocular pressure fluctuations in professional brass and woodwind musicians during common playing conditions. *Graefes Arch Clin Exp Ophthalmol*. 2011; 249: 895–901. 21234587 10.1007/s00417-010-1600-x

[25] Steinley D K-means clustering: a half-century synthesis. *Br J Math Stat Psychol*. 2006; 59: 1–34. 16709277 10.1348/000711005X48266

[26] Mignotte M A de-texturing and spatially constrained K-means approach for image segmentation. *Pattern Recogn Lett*. 2011; 32: 359–367.

[27] Dunn JC A fuzzy relative of the ISODATA process and its use in detecting compact well-separated clusters. *J Cybernetics*. 1973; 3: 32–57.

[28] Wang XY, Bu J A fast and robust image segmentation using FCM with spatial information. *Digit Signal Processing*. 2010; 20: 1173–1182.

[29] Cai W, Chen S, Zhang D Fast and robust fuzzy c-means clustering algorithms incorporating local information for image segmentation. *Pattern Recogn*. 2007; 40: 825–838.

[30] Li C, Huang R, Ding Z, Gatenby C, Metaxas D, Gore J A variational level set approach to segmentation and bias correction of images with intensity inhomogeneity. *Lect Notes Comput Sc*. 2008; 11: 1083–1091. 10.1007/978-3-540-85990-1_130 PMC2782702 18982712

[31] Kowalski CJ, Schneiderman ED, Willis SM PC program implementing an alternative to the paired t-test which adjusts for regression to the mean. *Int J Biomed Comput*. 1994; 37: 189–194. 7705901 10.1016/0020-7101(94)90117-1

[32] Wasserstein RL, Lazar NA The ASA statement on p-values: context, process, and purpose. *Am Stat*. 2016; 70: 129–133.

[33] Shrout PE and Fleiss JL Intraclass correlations: uses in assessing rater reliability. *Psychol Bull*. 1979; 86: 420–428. 18839484 10.1037//0033-2909.86.2.420

[34] McGraw KO, Wong SP Forming inferences about some intraclass correlation coefficients. *Psychol Methods*. 1996; 1: 390.

[35] Camras LJ, Stamer WD, Epstein D, Gonzalez P, Yuan F Differential effects of trabecular meshwork stiffness on outflow facility in normal human and porcine eyes. *Invest Ophthalmol Vis Sci*. 2012; 53: 5242–5250. 22786899 10.1167/iovs.12-9825

[36] Wang K, Johnstone MA, Xin C, et al. Estimating human trabecular meshwork stiffness by numerical modeling and advanced OCT imaging. *Invest Ophthalmol Vis Sci*. 2017; 58: 4809–4817. 28973327 10.1167/iovs.17-22175 PMC5624775

[37] Last JA, Pan T, Ding Y, et al. Elastic modulus determination of normal and glaucomatous human trabecular meshwork. *Invest Ophthalmol Vis Sci*. 2011; 52: 2147–2152. 21220561 10.1167/iovs.10-6342 PMC3080174

